# Le lymphome malin Hodgkinien au cours de la grossesse; quelle modalité thérapeutique: à propos d’un cas et une revue de la littérature

**DOI:** 10.11604/pamj.2018.30.20.14662

**Published:** 2018-05-09

**Authors:** Zenab Alami, Touria Bouhafa, Abderrahmane Elmazghi, Khalid Hassouni

**Affiliations:** 1 Service de Radiothérapie et Curiethérapie, CHU Hassan II, Faculté de Médecine et de Pharmacie de Fès, Maroc

**Keywords:** Lymphome de Hodgkin, grossesse, chimiothérapie, radiothérapie, Hodgkin lymphoma, pregnancy, chemotherapy, radiotherapy

## Abstract

Bien que le lymphome malin Hodgkinien ou (maladie de Hodgkin) ne représente que 10% de tous les lymphomes, c'est l'un des sous-types de lymphomes les plus communs diagnostiqués pendant la grossesse, en grande partie à cause de l'incidence maximale de la maladie qui coïncide avec l'âge de reproduction. La prise en charge d'une patiente enceinte atteinte d'une maladie de Hodgkin nécessite une approche multidisciplinaire, ainsi qu'une communication efficace avec le patient et sa famille. L'objectif est d'optimiser les chances de guérison de la mère tout en permettant l'accouchement d'un enfant en bonne santé. Nous rapportant un cas rare d'une patiente enceinte diagnostiquée pour une maladie de Hodgkin à 17 semaines d'aménorrhée.

## Abstract (en)

Malignant Hodgkin's lymphoma or (Hodgkin's disease) accounts for only 10% of all lymphomas but it is one of the most commonly diagnosed sub-types of lymphoma during pregnancy. This is due, in large part, to the maximum of disease incidence which coincides with reproductive age. The management of a pregnant patient with Hodgkin's disease requires multidisciplinary approach as well as effective communication with the patient and her family. The aim is to optimize mother's chances of being cured while ensuring the delivery of a healthy baby. We here report the rare case of a pregnant patient with Hodgkin's disease at 17 weeks of amenorrhea.

## Introduction

Bien que le lymphome malin Hodgkinien ne représente que 10 pour cent de tous les lymphomes, c'est l'un des sous-types de lymphomes les plus communs diagnostiqués pendant la grossesse, en grande partie à cause de l'incidence maximale de la maladie qui coïncide avec l'âge de reproduction. Cependant, l'association entre MDH et la grossesse est rare. La MDH est diagnostiqué dans environ 1/1000 à 1/6000 grossesses et représente 3 pour cent ou moins de tous les patients avec MDH [1]. Par conséquence, il existe peu de séries qui ont évalué les nombreux problèmes en matière de prise en charge chez ces patientes. Nous rapportant un cas rare d'une patiente enceinte diagnostiquée pour une maladie de Hodgkin à 17 semaines d'aménorrhée.

## Patient et observation

Nous rapportant le cas d'une patiente âgée de 28 ans primigeste à 17 semaines d'aménorrhée qui présentait une ADP cervicale isolée. La patiente ne rapportait pas de signes généraux tel que: une fièvre, sueurs nocturnes ou pertes de poids de plus de 10% durant les derniers 6mois. L'évaluation prénatale et l'échographie obstétricale étaient tout à fait normales. L'examen clinique a mis en évidence une ADP jugulo-carotidienne isolée faisant 2 à 3cm de diamétre. Un bilan biologique de routine a été réalisée, il a objectivé à la numération de formule sanguine un taux d'hémoglobine à 12,3g/dl, une hyperleucocytose à 15310 à prédominance Polynucléaire neutrophiles qui étaient à 9538/mm^3^ et des Lymphocytes à 4149/mmmm^3^. La VS était de 40mm. L'albuminémie, le taux sériques des LDH ainsi que le bilan hépatiques étaient normaux, Avec des sérologies: VIH, HVB, HVC, négatives. L'échocardiographie a mis en évidence une fonction ventriculaire normale avec un fraction d'éjection à: 69%. Une biopsie exérèse de l'adénopathie a été réalisée sous anesthésie locale. L'étude anatomopathologique a mis en évidence une Lymphadénopathie hodgkinienne de type scléronodulaire avec en immunohistochimie: CD30+, PAX5-, CD15-, CD20-, CD3-. L'évaluation radiologique s'est basée sur l'échographie qui était normale en dehors de l'ADP cervicale. La patiente a été classée stade IA selon la classification d'Ann Arbor La patiente a été mise sous chimiotéhrapie à la 19^éme^ semaine d'aménorrhée type ABVD (doxorubicin, bleomycin, vinblastine, et dacarbazine) 4 cycles toutes les 4 semaines. Une césarienne programmée a été réalisée à la 38^éme^ semaine après normalisation du bilan biologique. Le nouveau né était en bonne santé avec un poids normal à la naissance. Une TDM TAP a été réalisé en post partum objectivant une réponse complète. La patiente a bénéficié d'une radiothérapie conformationnelles sur l'aires GG cervicale Droit type IFRT (Invovled Field radiation therapy avec une dose totale de 30Gy. A 2 ans de la fin du traitement la patiente est en rémission complète avec un enfant en bonne santé.

## Discussion

La grossesse impose une limitation significative dans les possibilités de traitement de la maladie de Hodgkin (MDH). La santé de la mère et de l'enfant doivent être prises en considération ainsi que la survie à long terme de l'enfant s'il a été exposé durant la vie fœtale à un traitement à risque tératogène. Par conséquent, la décision pour démarrer le traitement chez une patiente enceinte est souvent difficile. Les caractéristiques de la maladie de hodgkin chez la femme enceinte sont identiques à la femme non-enceinte: la présentation clinique et les sous-types histologiques sont identiques chez les patientes de la même classe d'âge. Du point de vue histo-pathologique les caractéristiques sont les mêmes. Pour la stadification de la maladie de Hodgkin, la scanner ou le PET/Scanner cervical, Thoracique abdominale et pelvien sont typiquement demandé. Le scanner de l'abdomen ou du pelvis peuvent exposer le fœtus à une dose d'irradiation d'au moins 0,02Gy, qui est considérée potentiellement tératogène. Toutefois la séquence HASTE d'IRM a permis une imagerie meilleure et plus rapide et a largement remplacé l'IRM conventionnelle offrant ainsi les informations nécessaires sans risque d'irradiation sur le Fœtus. Pour cela, le scanner pelvien et abdominal doivent être évités durant la grossesse. Le PET Scanner doit être réalisé après l'accouchement pour évaluer la réponse thérapeutique. La stadification de la MDH durant la grossesse est basé sur l'histoire clinique, l'examen physique, les testes sanguins de routine et la biopsie osseuse, ainsi que l'IRM ou l'échographie qui peuvent être une bonne alternatives au scanner [2, 3]. La prise en charge thérapeutique doit prendre en considération la période gestationnelle, le stade de la maladie, la localisation et la progression des signes et des symptômes. Quand la maladie est diagnostiquée durant le deuxième ou le troisième trimestre, une chimiothérapie full dose peut être administrée sans augmentations de risques apparents de complications fœtales sévères. Durant le premier trimestre la chimiothérapie doit être évitée, l'exposition significative aux agents cytotoxiques durant les 4 premières semaines peut aboutir à un avortement spontané. Le risque de malformation congénitales augmente si l'exposition survient entre la 5^ème^ et le 12^éme^ semaine ce qui correspond à l'organogénèse. L'organogénèse est complète à la 12^éme^ semaine d'aménorrhée à l'exception du cerveau et des gonades. L'exposition à ces drogues durant le deuxième et le troisième trimestre n'est pas associé à un effet tératogène, mais peut aboutir à un retard de croissance intra-utérin, une prématurité ou une mortinatalité. De ce fait le suivi du bien-être fœtal pendant la grossesse est obligatoire. Une revue exhaustive de la littérature a conclu que l'ABVD (adriamycin, bleomycin, vinblastine, dacarbazine) est le régime de choix de chimiothérapie. Si l'indication de la chimiothérapie est posée l'ABVD semble être sans danger sur le développement fœtal quand il est utilisé durant le deuxième ou le troisième trimestre.

Une revue de la littérature a été conduite en 2008 par « American Society of Hematology Education Program ». Cette revue a évalué les papiers publiés entre 1950 et 2008 sur la base de donnée MEDLINE: elle a inclus 8 cas rapporté, 8 série de cas et 2 études cas-témoins. Parmi tous les cas analysés seulement un cas de malformation a été rapporté: dans ce cas la mère était traité avec de la cyclophosphamide orale durant les 3 trimestres de la grossesse et le nouveau avait une syndactylie [4]. L'expérience concernant le traitement avec le protocole MOPP (mechlorethamine, vincristine, procarbazine and prednisone) durant la grossesse est plus limités et à l'heure actuelle il n y a pas de traitement rapporté de femmes enceintes avec un protocole Stanford V ou avec un protocole BEACOPP renforcé. Pour les patientes avec une maladie avancée à un stade précoce de la grossesse, un retard de traitement peut affecter la survie. Par conséquent en ce basant sur l'analyse de facteur de risque, un traitement avec le protocole approprié de chimiothérapie (ABVD, BEACOPP) devrait être instauré rapidement et une interruption thérapeutique de la grossesse devrait être envisagé en raison des effets tératogènes potentiels de la chimiothérapie au cours du premier trimestre. Les patientes avec une MDH diagnostiquée au stade précoce au cours du premier trimestre peuvent faire l'objet d'un suivi à de courts intervalles pour détecter les signes de progression de la maladie sans aucun traitement jusqu'au deuxième trimestre. Plusieurs experts ont suggéré un traitement par mono chimiothérapie (de préférence Vinca alkaloids ou Anthracycline) pour ces patientes à faible risque. Un tel traitement est considéré comme sûr même pendant le premier trimestre, cependant, les données concernant son efficacité manquent. En outre, il n'est pas clair si un tel traitement peut induire une résistance à la chimiothérapie. Une monochimiothérapie peut également être envisagé chez les patientes diagnostiquées avec une MDH au cours du premier trimestre et qui refusent l'interruption thérapeutique de la grossesse en option. Dans tous les cas, au début du deuxième trimestre, un traitement adéquat par ABVD doit être démarré le plus tôt possible [5, 6].

Il faudrait envisager de reporter l'accouchement 2 à 3 semaines suivant le traitement pour permettre la récupération de la moelle ossuese, par ailleurs les bébés en période néonatale et spécialement les bébé prématuré ont des capacités limtés à métaboliser et éliminer les molecules de chimiothèrapie du fait d'une immaturité rénale et hépatique. En retardant l'accouchement après la chimiothérapie on permettra une élimintaion de ces molécules par voie placentaire [7]. Pour ce qui est de La Radiotéhrapie, quand elle est délivrée durant l'accouchement, l'exposition fœtale au rayonnement dépends de plusieurs facteurs incluant: la dose délivrée, la taille du champ de traitement et la distance entre la limite du champs de traitement et le fœtus. La protection abdominale peut d'avantage réduire l'exposition fœtale au rayonnement et doit toujours être utilisée. Généralement quand des doses conventionnelles de radiothérapie sont administrées, une distance de plus de 30cm de la limite du champs va limiter l'exposition fœtale à 4-20cGy. Par conséquent la radiothérapie peut être envisagée dans des circonstances spécifiques tel que des lymphomes limités à la région cervicale ou axillaire [8]. L'exposition à une dose entre 0,1-0,2Gy est considéré comme la dose limite aux malformations congénitales lorsqu'elle est délivré durant l'organogénèse. L'exposition au rayonnement durant le deuxième et le troisième trimestre est associée à un effet carcinogène qui peut inclure un risque élevé de développement de leucémie et de tumeurs solides durant la première décennie de vie (3-4 cas/1000 chez les exposés en intra-utérin aux rayonnements comparés à 2-3cas/1000 chez les non exposés. plus tard dans la vie ce sur-risque devient moins apparent ou même disparaît [5]. Un autre problème est le risque élevé de développer une pathologie neuro développementale, tel qu'une baisse du quotient intellectuel et parfois même un retard mental sévère [9]. L'exposition fœtale au rayonnement doit être mesuré individuellement et un physicien médical expert doit être consulté avant toute décision thérapeutique. Pendant de nombreuses années, on croyait que la grossesse augmentait les taux de rechute et de mortalité chez les patients atteints de HL Cependant, une étude cas-témoin de 48 cas de MDH associée à la grossesse a montré un taux de survie de 20 ans similaire à celui des témoins appariés. De plus, les nourrissons nés de femmes atteintes de MDH pendant la grossesse ne présentaient pas un risque plus élevé de prématurité ou de retard de croissance intra-utérin [10].

## Conclusion

L'approche diagnostique et thérapeutique d'une femme enceinte diagnostiquée avec une maladie de Hodgkin présente le défi de gérer deux vies. Le but est de donner à la mère les meilleures chances de guérison tout en préservant le développement sain du fœtus. La stratégie la mieux équilibrée est atteinte par une évaluation minutieuse de la Maladie de Hodgkin, particulièrement en ce qui concerne le stade de la maladie et l'âge gestationnel. Une approche multidisciplinaire est toujours nécessaire pour une meilleure prise en charge.

## Conflits d’intérêts

Les auteurs ne déclarent aucun conflit d'intérêts.
